# Social and public health implications of the legalisation of recreational cannabis: A literature review

**DOI:** 10.4102/phcfm.v11i1.2136

**Published:** 2019-11-19

**Authors:** Kebogile Mokwena

**Affiliations:** 1 Department of Social and Behavioural Health Sciences, Sefako Makgatho Health Sciences University, Pretoria, South Africa

**Keywords:** cannabis legislation, social outcomes, health outcomes, recreational use, legal system, injuries, Constitutional Court

## Abstract

**Background:**

After many years of legal struggles for the legalisation of recreational use of cannabis, the Constitutional Court of South Africa ruled in favour of the applicants in September 2018. Although the ruling issued caution regarding the social challenges accompanying this legalisation, it did not address how the country would deal with the societal consequences of this ruling.

**Aim:**

The aim of this article was to discuss the social and public health implications of the legalisation of recreational cannabis on South Africa.

**Methods:**

Literature review on the social, health and legal impacts of legalisation of cannabis, considering experiences of other countries that have legalised cannabis.

**Results:**

The legalisation brings a range of significant negative consequences, which include an expected increase in the number of users and the subsequent undesirable effects on the physical, mental and social health of communities.

**Conclusion:**

In terms of financial, infrastructural and human resources, South Africa cannot afford the consequences of the legalisation of recreational cannabis. Poor communities, children and the youth will carry the brunt of the scourge of cannabis use.

## Introduction

After a history of cannabis being outlawed for centuries, the Constitutional Court of South Africa ruled in favour of the legalisation of recreational cannabis in September 2018. Many South Africans, as well as government departments, view this ruling as a direct barrier to the goal of promoting health and well-being of the citizens, which is the mandate of public health programmes. Although many proponents of cannabis legalisation welcome the ruling, it is expected to trigger many consequences that will negatively influence health behaviour and health outcomes.^1^ The consequences are an increased demand on the health and social systems in the country, which had been under strain prior to this ruling. Cannabis use further contributes to poor adherence to antiretroviral therapy and management of HIV-related symptoms amongst HIV-infected individuals,^2^ as well as worsening of disease progression for TB-infected persons,^3^ which increases the burden of these two epidemics. The judgement further requires a legislative amendment to accommodate the change, and thus the need for policy makers and health service providers to consider the impact of this ruling, as well as the implications on the social and public health services.

Cannabis is the most commonly accessed illicit drug in South Africa,^4^ and the age of initiation of its use is reported to be between 11 and 12. The low price of cannabis enables widespread early initiation of use, low quit rate and a longer duration of use.^5^ The younger users are therefore vulnerable to disruptions in brain development, with long-term implications. The legalisation leads to an increase in the number of not only users, but also the under-age users and youth, who do not enjoy any specific protection despite their vulnerability.^6,7,8^ Moreover, as the number of users increases, so does the support and social acceptability, which diminishes the efforts to discourage its use.^9^

## Why the legalisation?

As in other countries, the legalisation of cannabis was driven by grassroots movements,^10^ with the main driver being the concept of self-determination, which argues that in a democracy the extent to which the state can be enabled to interfere and control personal aspects of the citizens’ lives is challenged. The self-determination concept includes behaviours that affect individuals’ health outcomes, as in the case of cannabis use. This argument was significantly used to challenge the outlawing of cannabis in many countries, including in South Africa. The agenda of cannabis legalisation is therefore driven by self-interest and self-determination, which do not necessarily support, promote or enable the principles of health promotion. Moreover, the current and past use of cannabis is more likely to be in favour of legalisation^9,11^ and often underestimates or even downplays its harmful effects.^12^ The consequences of legalisation of cannabis use therefore make it difficult to minimise the adverse effects of the drug.^13^

## Health and social impacts of legalisation of cannabis

The health arguments against the use of cannabis include its addictive nature,^14^ and that it is both a gateway and reverse gateway for hard drugs.^15^ It has also been directly linked to a range of adverse outcomes in physical health, which include lung cancer,^16^ impaired respiratory function, cardiovascular disease,^17^ elevated systolic blood pressure,^18^ stroke,^19^ mental disorders,^20,21,22^ which include schizophrenia, especially amongst young people,^23,24^ undesirable cognitive changes^25^ and disruption of normal brain development if used during adolescence.^26^

Cannabis has been reported to have adverse effects during pregnancy on both the mother and the baby.^27,28^ An offspring who is exposed to cannabis in utero is likely to engage in early indulgence of cannabis use.^29^ Cannabis use also contributes to a range of criminal activities.^30,31^ The educational and social impacts of cannabis use include poor academic performance and non-completion of studies,^32,33,34,35,36^ compromise in performing executive functions and challenges in social adjustment and vocational success.^37^ These challenges are likely to extend to the later life^38^ too and thus affect the ability to keep a job.^39,40,41^

Other researchers concluded that cannabis is associated with an increased frequency of both traffic^42,43^ and non-traffic injuries.^44^ With the high prevalence of violence and injuries in South Africa,^45,46^ it is reasonable to conclude that cannabis use contributes to many of the accidents, injuries and deaths on South African roads. This has been explained by cannabis use being associated with impaired driver cognition,^37,47,48,49^ psychomotor impairment and resultant car crashes.^50,51,52^

Cannabis use has also been associated with workplace injuries, where it presents with challenges for workplace productivity.^53,54,55,56^ Poor workplace productivity thus impacts on the economic performance of the country and is likely to increase with the legalisation of cannabis for recreational purposes. With the increasing rates of cannabis use, a relatively new clinical condition known as the Cannabinoid Hyperemesis Syndrome continues to be reported, which is characterised by episodes of nausea and vomiting, thus increasing evidence of the negative effect of cannabis on the gastrointestinal tract. A key symptom of this syndrome is a peculiar compulsive hot bathing pattern, which suggests an adverse effect on the central nervous system.^57,58,59,60^ However, the pathophysiology of this syndrome is not well understood and requires further investigation.

Cannabis use also increases the risk of poor mental health of the population, which can only be improved if the country makes the prevention and treatment of mental and substance use disorders a public health priority.^61,62^ However, this priority comes at a price that the country can hardly afford. Roadside testing for deterring driving after cannabis use is recommended,^63^ and this increases the demand for law-enforcement officers and technology to carry out such tests. It will also increase the proportion of people who depend on welfare.^64^ The increased demand also extends to services required to combat a range of health and social challenges, which include road traffic accidents, mental disorders as well as violence and severe crimes committed under the influence of substance abuse.^30,65^ Alcohol is by far the major substance of abuse, whereas cannabis is the most common illicit drug used, especially amongst youths, because it is easy to grow and cheap to buy.^66^

Although cannabis use continues to gain social acceptance, its association with potential adverse effects on pregnant women and their offspring poses a threat; thus, there is a need for specific interventions for this specific group of health-services recipients^28^ to combat the resultant adverse effects on pregnant women and their neonates.

Quitting cannabis use is difficult, expensive, takes a long time and is often unsuccessful. Once addicted, many users in South Africa need and want treatment services, but often have difficulty accessing such services.^67^ This results in substance abuse treatment utilisation being low amongst people from disadvantaged communities because of inequitable access to substance abuse treatment services.^68^ Barriers to treatment include stigma towards individuals with substance use disorders and negative beliefs about the quality and effectiveness of treatment. The scourge of cannabis use is therefore disproportionately borne by poor people who lack services for treatment and support, should they wish to quit.^69^

## Legalisation and potential increase in use

The impact of the legalisation of cannabis ruling will thus increase the demand and use, with associated social and health problems on both a short- and long-term basis, because of increased availability, greater social acceptance and possibly lower prices.^70,71^ The legalisation of recreational cannabis use is likely to increase both the amount of use amongst current users and increase the number of new users.^72,73^ Literature also shows that the impact of decriminalisation is concentrated amongst minors, who have a higher rate of uptake, especially in the period immediately after decriminalisation.^74^ The consequences of such an increase include a demand for legislative, health and social services, which can be human, infrastructural and financial, which are currently under strain.

## Legalised cannabis and the legal system

Although the current discussion on legalisation limits cannabis use to private homes, and children are meant to be protected from exposure, there are no specific measures to protect children who live in such homes. Such children are likely to be subjected to passive smoking of cannabis, with negative health outcomes, which include altered consciousness and even coma in some infants.^75,76,77,78^

The legalisation of cannabis is likely to further compromise the public security conditions in the country, as it will serve as a gateway to the use of harder drugs.^79^ The intended regulation for use, for example protecting minors, will be very difficult to implement, as strategies for such regulations are not in place. Currently, South Africa is already struggling to regulate the sales and use of alcohol and cigarettes, and an additional demand for the regulation of cannabis will increase the demand for regulation that is not likely to be met or implemented effectively without additional resources. There are therefore legal implications of this ruling, which require regulations that will prioritise public health over recreation by users and profits by cultivators and sellers of cannabis.^80^ This ruling does not, therefore, support efforts to prevent and reduce the harmful effects that result from the recreational use of cannabis, and South African policy-makers need to develop strategic and comprehensive controls to achieve minimum harm associated with cannabis use.

## The socio-economic impact of cannabis legalisation

Globally, poverty is associated with poor health-compromising behaviours, and cannabis dependence is greater amongst communities that are socio-economically compromised.^70,81^ The increase in the use of cannabis, which is a result of legalisation, will thus continue to increase the socio-economic disparity in South Africa, pushing more black people, who are more likely to use cannabis, to the lower end of the social class.^4,82^ Literature reports that in communities where substance abuse is rife, residents experience associated trauma, and they use addictive substances to cope with the negative psychological effects of trauma, resulting in a vicious cycle.^83^ Moreover, the Constitutional Court acknowledged that the legalisation of cannabis will result in a range of social problems, which the country needs to deal with. The ruling does not therefore address any remedies for the resultant social ills, which include mental health, crime and other challenges associated with the consequences of the ruling.

Although substance abuse in South Africa is high, there is lack of evidence-based interventions to combat the scourge. Currently, the country is battling with challenges to strengthen health systems to enable acceptable standards for ordinary healthcare services, and not much is streamlined towards the prevention of use of substances. The legalisation of cannabis thus comes at a time when its impact is likely to worsen the likelihood of channelling both human and financial resources towards prevention. The identification of vulnerable groups and interventions for prevention is currently much needed,^84^ and this need is expected to increase following the legalisation.

## Discussion

The status of cannabis as a gateway drug to a range of other illicit drugs use amongst individuals with mental disorders^85^ is a major concern for public health in South Africa. Although there have been arguments to emphasise the benefits of medical use of cannabis, such benefits are outweighed by the negative data, some of which are indicated in this article. Moreover, safe and effective alternative treatments are readily available, which can be given under medical advice. The legalisation of smoked cannabis is likely to cause significant public health risks, which will be a burden on the health system services in South Africa, and in the meantime pose a serious danger to a wide range of people.

The current increasing burden of mental illness has not been appropriately addressed, either in interventions to prevent or manage or treat the increasing numbers. The potential increase in the number of cannabis users, following this legalisation, is expected to worsen the situation as it increases the number of people who need treatment for addiction and other mental disorders that emanate from cannabis use. Cannabis use is associated with complications, considered to be serious because they lead to hospitalisation.^86^

One of the major public health challenges in South Africa is road accidents, specifically, driving under the influence of alcohol,^87,88^ which continues relentlessly despite various interventions by traffic officials and the police services. The situation is expected to increase significantly with cannabis use which, although incapacitates the user, cannot be easily detected.^89^ It is the view of some public health and behaviour scientists that the road accidents carnage needs to be approached as a public health matter, and not be considered a matter of policing or law enforcement.

The link between poverty and substance abuse^90,91,92^ is more pronounced amongst the poor who do not play any role in the fight for legalisation. The disparity between classes of people will thus be more pronounced than ever before.^82^ Even more concerning is the fact that the effects of cannabis use are passed on to future generations of the user.^93^

The legalisation of cannabis is expected to have an adverse impact on the health and social well-being of South Africans. The Constitutional Court, which ruled in favour of legalisation of cannabis, gave the parliament 2 years to amend the *Drugs and Drugs Trafficking Act* to accommodate this change. It is not clear how the parliament intends to approach this task to achieve minimum harm that results from the changes brought about by this ruling. The South African Ministries of Justice and Constitutional Development, Police, Health, Social Development, the National Director of Public Prosecutions, Doctors for Life and other Non Governmental Organisations (NGOs) that opposed the legalisation find themselves in a difficult position where they have to deal with the aftermath of a ruling that they did not support, a ruling which does not support or promote the health of the public.

Experiences from countries that legalised cannabis before South Africa include the increase in the uptake of the drug with resultant increase in road accidents and injuries. The legalisation, therefore, requires comprehensive strategies to keep the drug out of the reach of minors whilst increasing awareness and knowledge on the harmful effects of the drug. To get better insights on how to develop an appropriate framework to legalise marijuana, Canada should closely watch the development in its neighbouring country, the USA, where some of its states like Colorado, Oregon, Washington and Alaska have already legalised recreational use of marijuana.^94^

## Conclusion

In the USA, challenges of legalisation include an ineffective overarching federal regulatory structure, and an industry that seeks to exploit loopholes to maximise profit,^95^ which are the same issues that apply in South Africa. In industrialised countries like Canada, with adequate resources for prevention, management and treatment for cannabis addiction, legislation of cannabis has failed to protect the youth.^7^ The youth of South Africa are already vulnerable because of widespread prevalence of substance abuse, common ones being *nyaope*, ‘tik’ and cocaine, which the country has not been able to address satisfactorily. Lessons learnt from countries that have legalised cannabis indicate that South Africa cannot afford the costs of the consequences of the legalisation of cannabis, as it comes with demands on infrastructure, human, social, health services and financial resources brought about by a ruling of the highest court in the land. The poor and vulnerable communities, with the least programmes and resources for prevention and treatment, will continue to bear the brunt of the consequences of this ruling.
